# STAT5-regulated microRNA-193b controls haematopoietic stem and progenitor cell expansion by modulating cytokine receptor signalling

**DOI:** 10.1038/ncomms9928

**Published:** 2015-11-25

**Authors:** Nadine Haetscher, Yonatan Feuermann, Susanne Wingert, Maike Rehage, Frederic B. Thalheimer, Christian Weiser, Hanibal Bohnenberger, Klaus Jung, Timm Schroeder, Hubert Serve, Thomas Oellerich, Lothar Hennighausen, Michael A. Rieger

**Affiliations:** 1 LOEWE Center for Cell and Gene Therapy and Department of Medicine, Hematology/Oncology, Goethe University Frankfurt, Theodor-Stern-Kai 7, Frankfurt 60590, Germany; 2 Georg-Speyer-Haus, Paul-Ehrlich-Street 42-44, Frankfurt 60596, Germany; 3 Laboratory of Genetics and Physiology, NIDDK, National Institutes of Health, 9000 Rockville Pike, Bethesda, Maryland 20892, USA; 4 Department of Pathology, University Medical Center Göttingen, Robert-Koch-Street 40, Goettingen 37075, Germany; 5 Department of Medical Statistics, University Medical Center Göttingen, Humboldtallee 32, Goettingen 37073, Germany; 6 Department of Biosystems Science and Engineering, ETH Zurich, Mattenstrasse 26, Basel 4058, Switzerland; 7 German Cancer Consortium (DKTK), Heidelberg, Germany; 8 German Cancer Research Center (DKFZ), Im Neuenheimer Feld 280, Heidelberg 69120, Germany

## Abstract

Haematopoietic stem cells (HSCs) require the right composition of microRNAs (miR) for proper life-long balanced blood regeneration. Here we show a regulatory circuit that prevents excessive HSC self-renewal by upregulation of miR-193b upon self-renewal promoting thrombopoietin (TPO)-MPL-STAT5 signalling. In turn, miR-193b restricts cytokine signalling, by targeting the receptor tyrosine kinase c-KIT. We generated a miR-193b knockout mouse model to unravel the physiological function of miR-193b in haematopoiesis. *MiR-193b*^*−/−*^ mice show a selective gradual enrichment of functional HSCs, which are fully competent in multilineage blood reconstitution upon transplantation. The absence of miR-193b causes an accelerated expansion of HSCs, without altering cell cycle or survival, but by decelerating differentiation. Conversely, ectopic miR-193b expression restricts long-term repopulating HSC expansion and blood reconstitution. MiR-193b-deficient haematopoietic stem and progenitor cells exhibit increased basal and cytokine-induced STAT5 and AKT signalling. This STAT5-induced microRNA provides a negative feedback for excessive signalling to restrict uncontrolled HSC expansion.

## Abstract (web summary)

MicroRNAs regulate haematopoietic stem cell (HSC) development to ensure the correct generation of blood cells. Haetscher *et al*. show in mice that miR-193b controls the life-long self-renewal ability of HSCs via AKT and STAT5 pathways, with loss of miR-193b accelerating HSC expansion and reducing differentiation.

## Graphical abstract

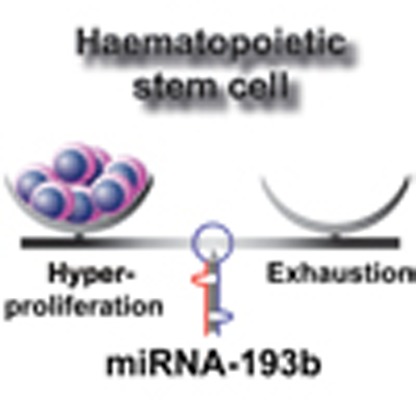

MicroRNAs (miRs) are small non-coding RNAs, which regulate gene expression by either degrading mRNAs or by inhibiting protein translation^1^. They simultaneously target various mRNAs and thereby fine-tune entire gene expression networks^1^. The necessity of miRs for normal long-term repopulating haematopoietic stem cell (LT-HSC) function became apparent by the haematopoietic-specific deletion of *Dicer*, an essential nuclease for the generation of mature miRs, leading to the loss of their life-long self-renewal ability^2^. MiR-125a and miR-125b support HSC maintenance, function and renewal^2^,^3^, and their upregulation may even drive leukaemogenesis^4^. Conversely, miR-126 impedes cell-cycle progression in LT-HSCs, and downregulation of miR-126 causes LT-HSC expansion^5^. Various miRs act at different haematopoietic developmental stages and lineages^6^, and altered miR expression may play a pivotal role in leukaemia onset and progression^1^,^7^,^8^.

We aimed to identify miRs induced by the self-renewal-promoting cytokine thrombopoietin (TPO)-mediated signalling in LT-HSCs. In particular, we assessed miRs downstream of TPO-induced activation of signal transducer and activator of transcription (STAT) 5A/B^9^. The STAT5A/B signalling pathway is activated by cytokine receptors, such as myeloproliferative leukemia (MPL), c-KIT and the receptors of interleukin 3, granulocyte–macrophage colony-stimulating factor and erythropoietin^10^. LT-HSCs require STAT5A/B activity for self-renewal and maintenance^11^. Constitutively active STAT5A/B promotes marked LT-HSC expansion and the subsequent development of a myeloproliferative syndrome^12^. Therefore, STAT5A/B signalling needs fine-tuning for normal LT-HSC self-renewal.

In this study, we identify miR-193b as a regulatory feedback molecule restricting excessive HSC self-renewal upon the activation of the self-renewal-promoting TPO-MPL-STAT5 signalling. To execute this function, miR-193b restricts cytokine signalling by targeting the tyrosine kinase c-KIT. In turn, miR-193b-deficient haematopoietic stem and progenitor cells (HSPCs) from miR-193b knockout mice exhibited increased basal and cytokine-induced STAT5 and AKT signalling, thereby promoting the consecutive expansion of HSCs. This STAT5-regulated miR balances cytokine signalling via the STAT5 and AKT pathways, providing a negative feedback for excessive signalling to restrict uncontrolled HSC expansion.

## Results

### *In vivo* expansion of LT-HSCs in the absence of miR-193b

**Figure 1:**
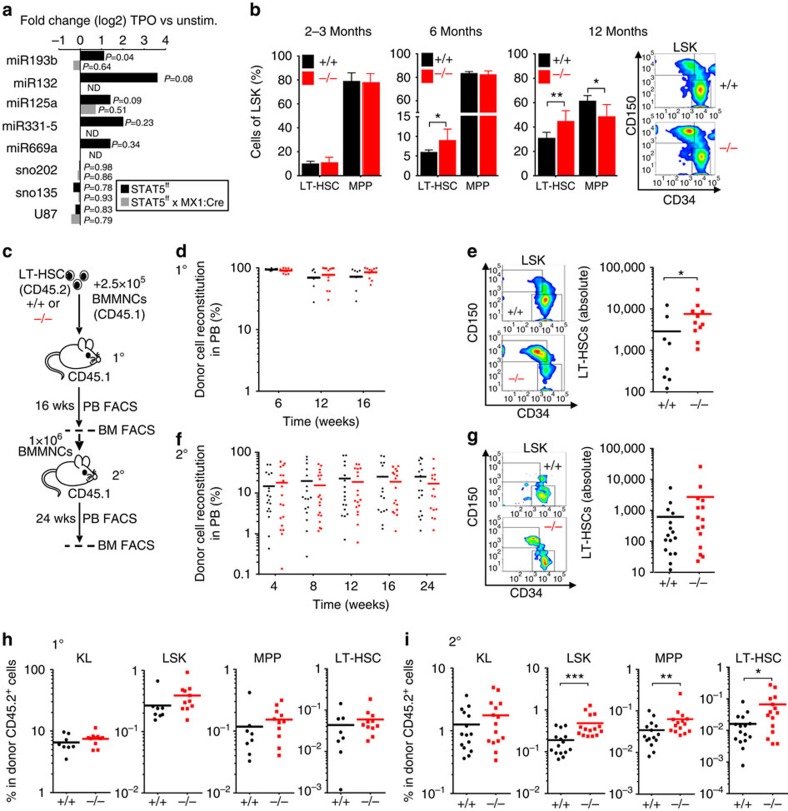
*In vivo* expansion of functional LT-HSCs in the absence of STAT5-regulated miR-193b. (**a**) Differential miR expression in the presence and absence of STAT5 after stimulation with TPO. Expression fold change was calculated and normalized to the corresponding untreated (unstim.) cells. The data represent the mean expression of two independent experiments using LT-HSCs from 16 to 20 mice per group. (**b**) Percentage of LT-HSCs and MPPs in the LSK fraction of the BM from mice of the indicated age groups, *N*=4 *miR-193b*^*+/+*^ mice and *N*=6 *miR-193b*^*−/−*^ mice at 2–3 months of age, *N*=6 mice/genotype at 6 months of age, *N*=8 *miR-193b*^*+/+*^ mice and *N*=4 *miR-193b*^*−/−*^ mice at 12 months of age. Exemplary FACS plots display the CD150 and CD34 expression of LSK (Lineage^−^ c-Kit^+^ Sca1^+^) BM cells from 12-month-old mice, representing LT-HSCs (CD150^+^ CD34^lo^) and MPPs (CD150^−^ CD34^+^). (**c**) Experimental scheme of the primary and secondary transplantation of LT-HSCs from *miR-193b*^*−/−*^and *miR-193b*^*+/+*^mice into NSG mice. (**d**) Donor cell engraftment in the peripheral blood of primary recipients after competitive transplantation of LT-HSCs from 12-month-old *miR-193b*^*−/−*^ and *miR-193b*^*+/+*^ mice was assessed via FACS. Mann–Whitney test. (**e**) FACS plots gated for donor LT-HSCs (left panel) and absolute numbers (right panel) after 16 weeks in both the femurs and tibiae of each primary recipient. Mann–Whitney test. (**f**) Donor cell engraftment in the peripheral blood of secondary recipients after transplanting 1 × 10^6^ BM cells from primary transplanted mice. BM of one primary transplanted mouse was transplanted into two recipients. Mann–Whitney test. (**g**) FACS plots gated for donor LT-HSCs (left panel) and absolute numbers (right panel) after 24 weeks in both femurs and tibiae of each secondary recipient. Mann–Whitney test. (**h**,**i**) The distribution of donor stem and progenitor populations in primary (**h**) and secondary (**i**) recipient BM. Mann–Whitney test. The data are represented as the mean±s.d. *P<0.05; **P<0.01 and ***P<0.001.

To identify miRs that are extrinsically regulated by the self-renewal-promoting signalling axis comprising TPO, its receptor MPL and the transcription factors STAT5A/B, we compared miR expression patterns in LT-HSCs of STAT5A/B-deficient and wild-type (WT) control mice^13^ that were stimulated with TPO or kept unstimulated, by quantitative PCR (qPCR; Fig. 1a). The differential miR pattern revealed five miRs that were >2-fold upregulated by TPO only in the presence of STAT5A/B: miR-193b, miR-132, miR-125a, miR-331-5p and miR-669a (Fig. 1a and Supplementary Data 1). We focused on the function of the intergenic miR-193b in haematopoiesis, because miR-193b is selectively expressed in LT-HSCs and to a lesser extend in multipotent progenitors (MPPs), but not in lineage-committed progenitors and mature blood cells, as shown by us (Supplementary Fig. 1a) and others^3^,^6^. Furthermore, haematopoietic stress induced by the cytokine storm 10 days after 5-fluorouracil (5-FU) treatment upregulated miR-193b expression in LT-HSCs (about 2.5-fold in comparison to steady-state). Although the induction of miR-193b expression was even more pronounced in lineage-committed progenitors and mature blood cells than in LT-HSCs caused by 5-FU treatment, the expression level in these committed cells was still 1,000 times lower than in LT-HSCs (Supplementary Fig. 1b). Recently, we demonstrated that STAT5A/B binds to the miR-193b promoter in the murine mammary gland^14^. Here we could show that STAT5A/B is required for the cytokine-induced miR-193b transcription in LT-HSCs.

To unravel the function of miR-193b in haematopoiesis, we generated miR-193b knock-out mice, which were viable without visible abnormalities^15^. First we investigated the steady-state haematopoiesis of 2- to 3-month-old *miR-193b*^*−/−*^ mice. Compared with WT mice, no significant differences (according to *t*-tests) were observed in the mature blood cell lineages in peripheral blood, bone marrow (BM) or spleen of *miR-193b*^*−/−*^ mice (Supplementary Fig. 2a–c). The percentage and number of defined BM progenitor cells were also unchanged (Fig. 1b and Supplementary Fig. 2d,e). However, *miR-193b*^*−/−*^ mice over 6 months of age displayed an unexpected increase in LT-HSCs in the LSK (Lineage^−^Sca1^+^c-KIT^+^) compartment (Fig. 1b), whereas total LSK cell numbers were not altered (Supplementary Fig. 2e). The accumulation of LT-HSCs increased with age, as 1-year-old mice showed a 1:1 ratio of LT-HSCs and MPPs (Fig. 1b). Yet, we only determined the LT-HSC frequency by their well-established marker phenotype, but we needed to confirm their true identity by their long-term blood reconstitution ability. To corroborate that *miR-193b*^*−/−*^ LT-HSCs were fully functional, we performed a competitive transplantation of LT-HSCs from 1-year-old miR-193b-deficient or WT mice into recipients and then monitored donor blood reconstitution (Fig. 1c). The miR-193b-deficient LT-HSCs reconstituted equally well as WT LT-HSCs (Fig. 1d) and exhibited normal production of T, B and myeloid cells (Supplementary Fig. 2g). Strikingly, when we analysed the distribution of LT-HSC and progenitor cells in primary recipient BM, we determined a more than twofold increase in phenotypic LT-HSC numbers in the absence of miR-193b in comparison to the WT controls (Fig. 1e). Although donor cell engraftment in the BM was only slightly enhanced in the absence of miR-193b (Supplementary Fig. 2f), overall BM donor cellularity was markedly increased, thereby suggesting that *miR-193b*^*−/−*^ LT-HSCs self-renew extensively after transplantation stress to repopulate the recipient (Supplementary Fig. 2h). We further challenged the self-renewal ability of miR-193b-deficient LT-HSCs by transplanting unfractionated BM cells from primary recipients into secondary recipient mice. Again, both the WT and knockout group reconstituted the secondary recipients almost equally well, which clearly indicated that miR-193b-deficient LT-HSCs were fully functional (Fig. 1f). When we gated for LT-HSCs in the BM of secondary recipients, we again measured an increased proportion and number of LT-HSCs in those recipients that received *miR-193b*^*−/−*^ cells (Fig. 1g). Assessing the donor-derived HSPC distribution in primary and secondary recipient BM, we observed a consistent increase in *miR-193b*^*−/−*^ LT-HSCs and subsequent increase in LSK cell numbers (Fig. 1h,i). Although no difference was found in the primary recipients of either *miR-193b*^*−/−*^ or *miR-193b*^*+/+*^ LT-HSCs (Fig. 1h), secondary recipient BM accumulated *miR-193b*^*−/−*^ LT-HSCs and LSK cells (Fig. 1i). This proves that the self-renewal of LT-HSCs leading to an enlarged compartment of competent LT-HSCs is intrinsically promoted in the absence of miR-193b. Of note, the recipients that received the *miR-193b*^*−/−*^ donor cells displayed a slightly lower donor cell chimerism in the BM (Supplementary Fig. 2f,h), indicating that although they received more LT-HSCs from primary recipients and again showed an enhanced self-renewal and expansion of LT-HSCs, the ability of these LT-HSCs to produce the same output on mature cells seemed altered. Whether this is due to a delay in differentiation of LT-HSCs or an altered clonal fitness remains to be clarified.

### More miR-193b-deficient LT-HSCs are in active cell cycle

**Figure 2:**
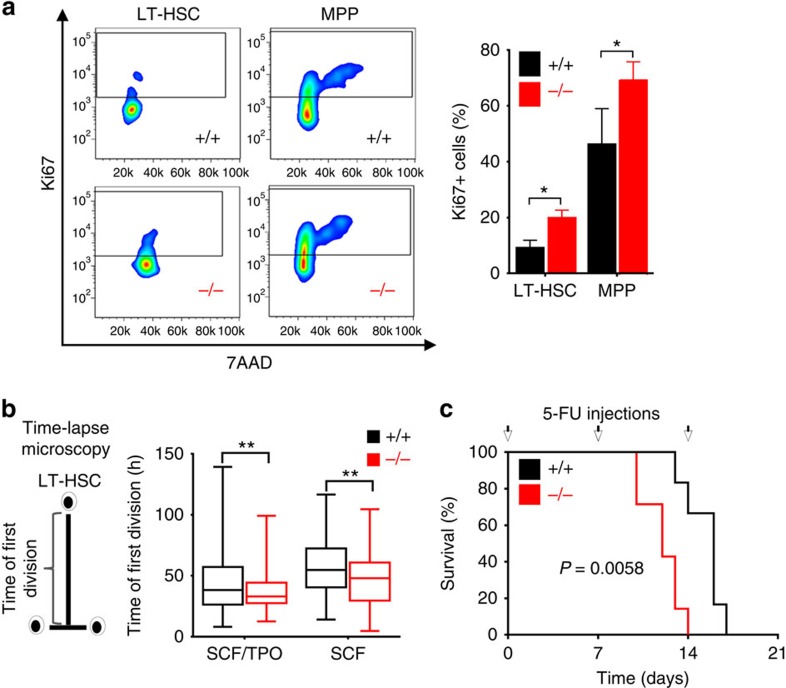
An increased number of miR-193b-deficient LT-HSCs are in active cell cycle. (**a**) Exemplary FACS plots and quantification of Ki67 and 7-AAD (DNA content) of LT-HSCs and MPPs to assess quiescent and cycling cells. (**b**) Time of the first division of LT-HSCs determined by video-microscopy-based cell tracking. The box plots represent the median with whiskers min to max. (**c**) A Kaplan–Meier survival curve of the mice injected with 5-fluorouracil (5-FU) at the indicated time points (arrows). *N*=7 *miR-193b*^*−/−*^ mice, *N*=6 *miR-193b*^*+/+*^ mice. Gehan–Breslow–Wilcoxon test. All data are represented as the mean±s.d. *P<0.05; **P<0.01.

Most LT-HSCs are quiescent (G0 phase) in homeostasis. The expansion of the LT-HSC population may indicate that an increased proportion of LT-HSCs are actively cycling in the absence of miR-193b. Therefore, we examined the cell-cycle phases in 2- to 3-month-old mice (Fig. 2a). Indeed, there were a significant higher proportion of LT-HSCs and MPPs in cell-cycle (Ki67^+^) in *miR-193b*^*−/−*^ mice (Fig. 2a). The continuous observation of individual LT-HSCs by time-lapse microscopy-based cell tracking^16^,^17^ allowed us to determine the time of entry into the first division once cells were exposed to *in vitro* culture. The *miR-193b*^*−/−*^ LT-HSCs displayed an earlier time point of division compared with WT controls under minimal (stem cell factor (SCF) only) and self-renewal-promoting (TPO and SCF) cytokine conditions (Fig. 2b). Next, we functionally confirmed these findings by repetitively treating mice with 5-FU, which eliminates proliferating cells. Quiescent LT-HSCs only become susceptible to 5-FU during haematopoietic stress, driving them into cycle. We injected 5-FU into *miR-193b*^*−/−*^ and *miR-193b*^*+/+*^ mice once a week, until the mice suffered from fatal haematopoietic failure (Fig. 2c). Although most of the WT mice died after the third round of 5-FU injections, all miR-193b-deficient mice died already after two subsequent injections, most likely because the hyperproliferative LT-HSCs were quickly extinguished (Fig. 2c). These results show at the phenotypic, molecular and functional level that the absence of miR-193b leads to an intrinsically controlled expansion of fully functional LT-HSCs, as they exhibit reduced quiescence and increased self-renewal.

### MiR-193b controls HSPC expansion by altering differentiation

**Figure 3:**
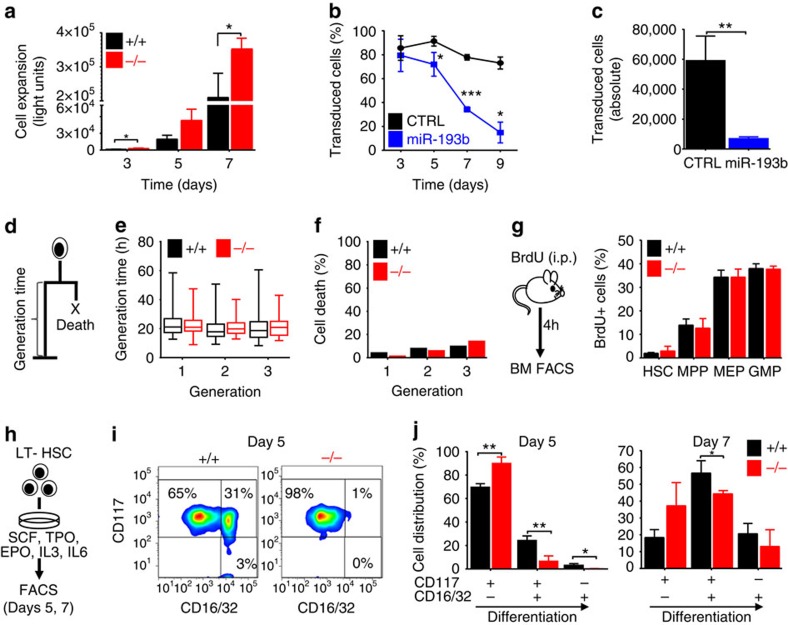
MiR-193b controls HSPC expansion by altering differentiation and not by influencing cell cycle or survival. (**a**) Proliferation assay of sorted LT-HSCs in serum-free liquid culture (SCF, TPO, IL3, IL6 and EPO). *N*=3 independent experiments. (**b**,**c**) Lentiviral transduction of LT-HSCs with miR-193b or CTRL vectors and cell culture (SCF, TPO). *N*=3 independent experiments. (**b**) The percentage of transduced cells was determined via FACS. (**c**) Absolute number of cells after 9 days in culture. (**d**) Generation time and cell death determined using video-microscopy-based cell tracking. (**e**) Generation times in subsequent generations of LT-HSCs determined via single-cell tracking. (**f**) Cell death events assessed via single-cell tracking. (**g**) BM FACS analysis of BrdU incorporation in HSPC populations after 4 h of *in vivo* BrdU pulse. *N*=3 mice per genotype. (**h**) Experimental scheme for the *in vitro* differentiation assay. (**i**) Exemplary FACS plots of CD16/32/CD117 expression at day 5 of differentiation. (**j**) Cell distributions at days 5 and 7 determined according to their CD117 and CD16/32 expression. *N*=3 individual mice per genotype. All data are represented as the mean±s.d. *P<0.05; **P<0.01 and ***P<0.001.

We then assessed the immediate consequences of the absence of miR-193b on the function and fate of LT-HSCs. First, we measured the expansion of LT-HSCs in liquid culture (Fig. 3a) and found that LT-HSCs from *miR-193b*^*−/−*^ mice gave rise to a twofold increase in cell numbers over a 7-day-period compared with WT LT-HSCs (Fig. 3a). Conversely, lentiviral expression of miR-193b in LT-HSCs led to a dramatic reduction of miR-193b-expressing progeny over time, whereas the percentages of control vector transduced cells or of cells lentivirally expressing the unrelated miRs-132/212 remained largely constant (Fig. 3b,c, and Supplementary Figs 3a and 4a). Of note, ectopic expression of miR-193b in GMPs did not influence their expansion, and the percentage of transduced cells did not change over time (Supplementary Fig. 4b), indicating a distinct function of miR-193b in LT-HSCs. The increased expansion of LT-HSCs and their progeny leading to elevated levels of mature blood cells may be explained by increased proliferation, reduced apoptosis or delayed differentiation. To dissect these various cell fates at a single-cell resolution, we monitored the individual LT-HSCs and their progeny of miR-193b-deficient and WT mice using video-microscopy-based cell tracking (Fig. 3d and Supplementary Fig. 5). Interestingly, the absence of miR-193b had no influence on cell-cycle duration in dividing LT-HSCs and their progeny for many generations (Fig. 3e). Moreover, there was no decrease of cell death events in the absence of miR-193b (Fig. 3f). To verify that the absence of miR-193b does not influence the cell-cycle duration of HSPCs, we determined HSPC proliferation *in vivo* by pulsing *miR-193b*^*−/−*^ and *miR-193b*^*+/+*^ mice with 5-bromodeoxyuridine (BrdU) for 4 h. Accordingly, there was no difference in the cell-cycle distribution in LT-HSCs, MPPs, GMPs or MEPs, which further confirmed that the absence of miR-193b does not shorten the cell cycle of HSPCs (Fig. 3g). These results show that the absence of miR-193b increases the number of LT-HSCs in active cell cycle (Fig. 2), but does not alter the cell-cycle progression of proliferating LT-HSCs or their progeny during steady-state haematopoiesis (Fig. 3).

We asked whether *miR-193b*^*−/−*^ LT-HSCs show a delayed differentiation that causes an accumulation of highly proliferative progenitors. Therefore, we determined distinct differentiation stages of LT-HSCs that were cultured for several days via fluorescence-activated cell sorting (FACS; Fig. 3h). After 5 days of culture, 25% of WT cells expressed markers similar to GMP-like cells (CD117^+^, CD16/32^+^). Meanwhile, most miR-193b-deficient cells remained more immature (CD117^+^, CD16/32^−^; Fig. 3i,j). Overall, the increased expansion of cells from *miR-193b*^*−/−*^ LT-HSCs was due to a delay in differentiation and not a change in proliferation or survival.

### Increased cytokine signalling in miR-193b^*−/−*^ LT-HSCs

**Figure 4:**
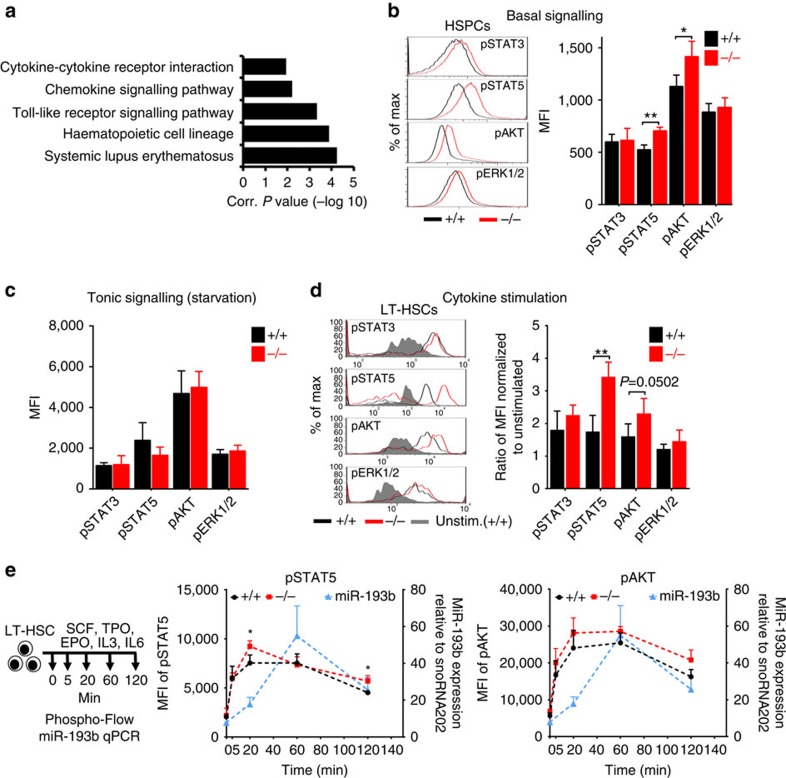
The absence of miR-193b increases basal and cytokine-stimulated signalling in LT-HSCs. (**a**) Functional annotation analysis using DAVID (KEGG pathways) of the upregulated and downregulated genes in sorted LSK cells derived from *miR-193b*^*−/−*^ and *miR-193b*^+/+^ mice determined via RNA sequencing. Only clusters with a score >1 are displayed. (**b**) Representative histograms and quantification of phosphoflow cytometry analysing basal signalling pathways of HSPCs from *miR-193b*^*−/−*^ and *miR-193b*^+/+^ mice. *N*=3 independent experiments. (**c**) Equal signalling intensities were observed in sorted LT-HSCs after 1 h of starvation (tonic signalling) as assessed by phosphoflow cytometry. *N*=4 independent experiments. (**d**) Representative histograms and quantification of phosphoflow cytometry of LT-HSCs stimulated with a myeloid cytokine cocktail for 20 min after starvation. The mean fluorescence intensity (MFI) was normalized to starved and unstimulated cells. *N*=4 independent experiments. (**e**) Quantification pSTAT5 and pAKT via phosphoflow cytometry and miR-193b expression via qPCR in LT-HSCs stimulated with a cytokine cocktail at various time points. The miR-193b expression was normalized to snoRNA202. *N*=3 mice per group (phosphoflow cytometry) and *N*=3 individual experiments (qPCR). All data are represented as the mean±s.d. *P<0.05; **P<0.01.

Next, we aimed to assess the molecular mechanism by which miR-193b restrains HSPC expansion and LT-HSC self-renewal. We performed RNA sequencing of LSKs from *miR-193b*^*−/−*^ and *miR-193b*^*+/+*^ mice to elucidate differences in the gene expression profile. Using a regulation threshold of 1.5-fold (*P*<0.05), we identified 41 upregulated and 117 downregulated genes in the absence of miR-193b (Supplementary Fig. 6 and Supplementary Data 2). Kyoto Encyclopedia of Genes and Genomes (KEGG) pathway analysis of these regulated genes using DAVID^18^ and Ingenuity Pathway Analysis suggested the involvement of altered cell signalling (Fig. 4a and Supplementary Data 3 and 4). This result guided us to investigate major signalling pathways that are activated by cytokines, such as Janus kinase/STAT, PI3K/AKT (phosphatidylinositol 3-kinase/thymoma viral proto-oncogene) and MAPK/ERK (mitogen-activated protein kinase). Phosphoflow cytometry of phosphorylated STAT3, STAT5, AKT and ERK revealed an increase in the basal signalling levels of STAT5 and AKT in *miR-193b*^*−/−*^ BM cells (Fig. 4b). The hyperactivation of signal transduction, particularly of STAT5 (ref. 12), may lead to the observed expansion of LT-HSCs in miR-193b-deficient mice. Therefore, we assessed tonic and cytokine-activated signalling in LT-HSCs using phosphoflow cytometry. Although tonic signalling levels after cytokine starvation were similar (Fig. 4c), STAT5 and AKT phosphorylation were 2- and 1.5-fold stronger after cytokine stimulation, respectively, in miR-193b-deficient LT-HSCs compared with WT counterparts (Fig. 4d). Next, we determined the kinetics of STAT5 and AKT signalling in temporal relation to miR-193b expression in LT-HSCs upon cytokine stimulation, and investigated changes in STAT5 and AKT signalling in the absence of miR-193b (Fig. 4e). After a rapid induction already at 5 min of stimulation, the levels of pSTAT5 and pAKT further increased to a maximum at 20 min in LT-HSCs from *miR-193b*^*+/+*^ and *miR-193b*^*−/−*^ mice. pSTAT5 and pAKT levels remained constant for 60 min before declining again at 120 min. Intriguingly, *miR-193b*^*−/−*^ LT-HSCs showed an overshooting activation of pSTAT5 at 20 min, that did not decline as much as the pSTAT5 levels in *miR-193b*^*+/+*^ LT-HSCs at 120 min (Fig. 4e). *MiR-193b*^*−/−*^ LT-HSCs showed a higher induction of pAKT at 20 min, which remained above the pAKT levels of *miR-193b*^*+/+*^ LT-HSCs until 120 min (Fig. 4e). The expression of miR-193b was instantly induced by cytokine stimulation and showed the strongest expression at 60 min after stimulation before it already declined at 120 min. Importantly, already 20 min after stimulation there was a twofold increase in miR-193b expression in comparison to unstimulated LT-HSCs (Fig. 4e).

These results underline that the cytokine-induced activation of STAT5 leads to the rapid upregulation of the miR-193b as a STAT5-dependent miR, and the time shift between maximum pSTAT5 (at 20 min) and maximum miR-193b expression (at 60 min) would be expected from a transcribed target gene of STAT5. However, in the absence of miR-193b there is an overshooting pSTAT5 and pAKT activation signal, which would have been dampened in the presence of the already increased miR-193b levels at 20 min. Furthermore, the activation of STAT5 and AKT persisted longer in the absence of miR-193b, which suggests a negative regulation of the signalling kinetics by the miR-193b.

### MiR-193b targets c-KIT and thereby modulates signalling

**Figure 5:**
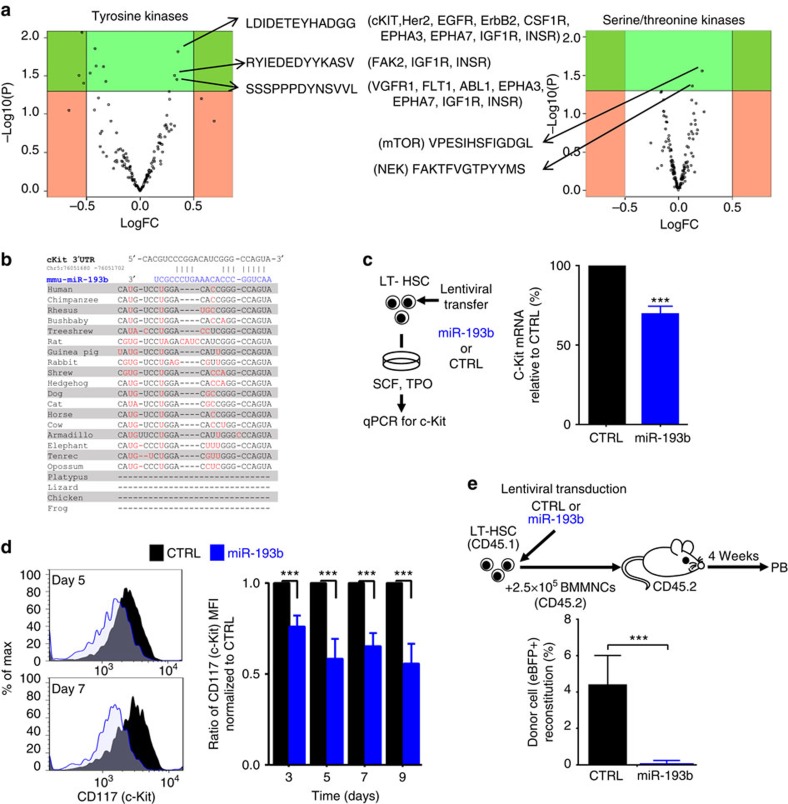
MiR-193b targets c-KIT expression and thereby modulates signalling in HSPCs. (**a**) PamGene array volcano plots of tyrosine and serine/threonine kinases in BM cells derived from *miR-193b*^*−/−*^ and *miR-193b*^+/+^ mice. Altered peptides and potential kinases (in brackets) are shown. *N*=4 independent experiments. (**b**) Target scan analysis of the 3′-untranslated region of murine *c-Kit*, including the location and conservation of the miR-193b-binding site. Sequence differences between the species are highlighted in red. (**c**) c-Kit mRNA expression upon miR-193b ectopic expression in LT-HSCs via qPCR. C-Kit mRNA expression was normalized to Gpdh mRNA. Transduction efficiencies were 94% and 90% for control and miR-193b, respectively. *N*=3 independent experiments. (**d**) FACS analysis of c-KIT surface expression upon miR-193b ectopic expression in LT-HSCs via FACS. A representative FACS plot and relative quantification of the c-KIT expression normalized to the CTRL (control) transduced cells are displayed. *N*=3 independent experiments. (**e**) Competitive transplantation of LT-HSCs that were lentivirally transduced with either miR-193b or CTRL 24 h prior transplantation. The transduction efficiency was 26% in both groups. Donor cell engraftment was measured in the peripheral blood after 4 weeks. *N*=5–6 mice per group. Mann–Whitney test. All data are represented as the mean±s.d. ***P<0.001.

The increased signalling output may be caused by kinase hyperactivation. Therefore, we applied PamGene array technology to quantitatively compare the activity of hundreds of tyrosine and serine/threonine kinases in the BM cells of *miR-193b*^*−/−*^ and *miR-193b*^*+/+*^ mice in an unbiased way. Overall, we did not observe global changes in tyrosine or serine/threonine phosphorylation patterns in miR-193b-deficient cells. However, we clearly determined specific changes in a small group of peptides (Fig. 5a and Supplementary Data 5). One peptide found to be hyperphosphorylated was a target of c-KIT, the receptor of the cytokine SCF and an essential receptor tyrosine kinase in haematopoiesis. Altered c-KIT signalling has detrimental effects on LT-HSC biology^19^,^20^,^21^. Another hyperphosphorylated peptide was a target of the serine/threonine kinase mTOR (mechanistic target of rapamycin), which acts downstream of PI3K/AKT signalling. Interestingly, PI3K/AKT signalling was enhanced in *miR-193b*^*−/−*^ HSPCs at basal levels and after cytokine stimulation.

**Figure 6:**
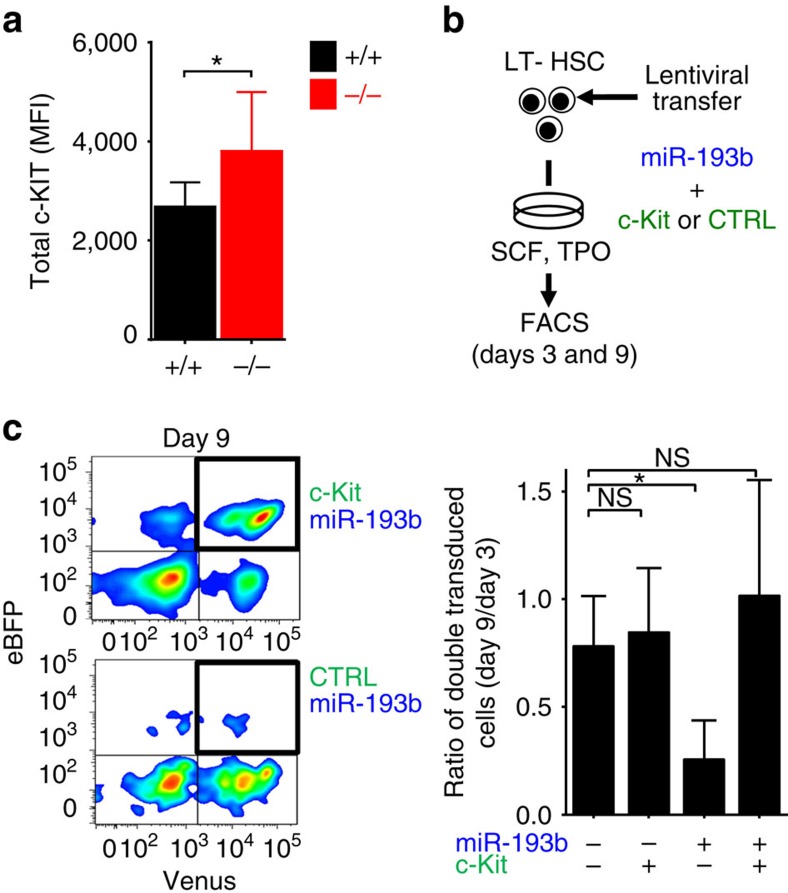
Ectopic c-Kit expression can rescue miR-193b-mediated effects in HSPCs. (**a**) Endogenous c-KIT protein expression in BM cells of *miR-193b*^*−/−*^ and *miR-193b*^+/+^ mice measured via FACS. *N*=4 mice per group. (**b**) Rescue of HSPC survival by ectopic c-KIT expression. Re-expression of c-KIT lacking the 3′-untranslated region in miR-193b-transduced LT-HSCs via lentiviral co-infection and culture for several days. (**c**) Representative FACS plots and quantification of cultured cells at day 9. Ratio of double transduced cells (day 9/day 3) displayed as the mean±s.d. *N*=3 independent experiments. All data are represented as the mean±s.d. MFI, mean fluorescence intensity. *P<0.05; NS, not significant.

As the *c-Kit* mRNA contains a predicted conserved miR-193b target sequence (Fig. 5b), and c-KIT activity was elevated in the absence of miR-193b, we predicted that miR-193b can modulate c-KIT expression in HSPCs. Indeed, there was a 30% reduction of c-Kit mRNA expression in LT-HSCs ectopically expressing miR-193b (Fig. 5c). More importantly, we determined a 40% reduction of c-KIT surface expression on HSPCs that expressed miR-193b in comparison to control vector transduced cells, measured by FACS (Fig. 5d). Because LT-HSCs with diminished c-KIT function are severely impaired in recipient repopulation^19^,^20^, we assessed the blood reconstitution of miR-193b-expressing LT-HSCs after transplantation. As hypothesized, no donor cell reconstitution was detected in mice transplanted with LT-HSCs ectopically expressing miR-193b (Fig. 5e). Reduced cell expansion and lack of blood reconstitution following transplantation with LT-HSCs overexpressing miR-193b resembles the phenotype of LT-HSCs harbouring dysfunctional c-KIT^19^,^20^. Next, we assessed the c-KIT protein expression in *miR-193b*^*−/−*^ BM cells and showed via FACS that miR-193b-deficient cells expressed 30% more c-KIT protein in comparison to their respective *miR-193b*^*+/+*^ counterparts (Fig. 6a), indicating that the absence of miR-193b leads to higher c-KIT protein levels.

To consolidate that the observed phenotype in miR-193b-expressing LT-HSCs was at least partly due to the reduced c-KIT expression, we lentivirally expressed both *c-Kit* lacking the miR-193b target site (Supplementary Fig. 3b) and miR-193b in LT-HSCs (Fig. 6b). We then assessed the expansion of double-transduced cells in culture. Although cells expressing miR-193b and a control vector nearly disappeared over the course 7 days, miR-193b-expressing cells co-transduced with c-KIT expanded in culture, thereby rescuing the miR-193b-mediated effect (Fig. 6c). These results show that STAT5-activated miR-193b regulates c-KIT expression, probably among other important factors, and thereby subsequently influences signalling networks to control the fate of LT-HSCs.

## Discussion

Two prominent signalling pathways guiding LT-HSC self-renewal and proliferation, STAT5A/B and PI3K/AKT, are hyperactivated in the absence of miR-193b leading to a more active LT-HSC population that expands over time (Supplementary Fig. 7). Constitutive active STAT5 signalling induces LT-HSC expansion and finally results in a myeloproliferative disease in mice^12^. Furthermore, many leukaemogenic alterations (for example, mutated *c-Kit*, *Flt3-ITD* and *BCR-ABL*) require chronically high STAT5 activation for disease development^22^,^23^,^24^. PI3K/AKT signalling promotes cell proliferation and survival^25^, and hyperactivation of this pathway results in LT-HSC expansion and exhaustion, and in the development of leukaemia^26^. Furthermore, SCF is essential for the survival and proliferation of all HSPCs, and it transmits its signal after binding to c-KIT leading to STAT5, PI3K/AKT and ERK signalling^21^. Activating c-KIT mutations, which are common in acute leukaemias, cause SCF-independent constitutive, dysregulated signalling and eventual uncontrolled expansion of leukaemic blasts^27^. Conversely, in mouse models, hypomorphic c-KIT mutations compromise LT-HSCs, which can easily be outcompeted by WT LT-HSCs in transplantations without harsh conditioning^28^. STAT5 and AKT signalling need to be in tight balance^26^, and are controlled by negative regulators such as suppressor of cytokine signaling (SOCS) and phosphatase and tensin homolog (PTEN)^10^,^29^,^30^,^31^,^32^. PTEN-deficient LT-HSCs display increased PI3K/AKT signalling and a hyperproliferative phenotype with long-term exhaustion^29^,^30^,^32^. In this study, we found that miR-193b is an important negative regulator of basal and cytokine-stimulated signalling and hyperactivation in LT-HSCs. Therefore, as miR-193b expression is triggered by STAT5 signalling, this axis represents a classical negative feedback mechanism. Strikingly, although *miR-193b*^*−/−*^ LT-HSCs were more actively cycling and expanding, they did not exhaust and remained fully functional. This aspect distinguishes miR-193b function from other haematopoietic negative regulators, such as PTEN, thereby suggesting that miR-193b is able to fine-tune HSC numbers. The tolerated expansion of LT-HSCs over time might also be supported by our findings that the absence of miR-193b does not change the cell-cycle length in proliferating HSPCs, but rather regulates the decision of active cell-cycle entry versus quiescence.

It is not surprising that miR-193b is downregulated in leukaemia and other cancer entities^7^,^33^,^34^,^35^,^36^. Although we did not observed increased cancer incidence in 1-year-old *miR-193b*^*−/−*^ mice, it would be intriguing to test whether the absence of miR-193b cooperates with known oncogenes. Especially, the initiation of pre-cancerous (stem) cells at an early disease stage might be supported by enhanced self-renewal in the absence of the tumour-suppressing miR-193b, as STAT5 activation plays a key role in establishing pre-cancerous clonal dominance in stem cells^37^,^38^. We recently reported the expansion of mammary epithelial stem cells in the absence of miR-193b^14^, which suggests a general miR-193b function in restricting adult stem cell proliferation. Further evaluation is warranted to determine whether miR-193b downregulation is an early event in tumourigenesis.

## Methods

### Mice

Male and female C57BL/6, B6.SJL-PtprcaPepcb/BoyJ, NOD.Cg-Prkdcscid Il2rgtm1Wjl/SzJ (referred to as NSG), B6.129S6-Stat5a/Stat5btm2Mam/Mmjax (referred to as STAT5^fl/fl^)^13^ and B6.129S6-Stat5a/Stat5btm2Mam/Mmjax x B6.Cg-Tg(Mx1-cre)1Cgn/J (referred to as STAT5^fl/fl^ x Mx1::Cre)^13^ mice were purchased from Jackson Laboratory or bred in our animal facility. Male miR-193b-deficient (referred to as *miR-193b*^*−/−*^) and corresponding WT littermate control mice (referred to as *miR-193b*^+/+^)^15^ were used in this study. The mice were 8–14 weeks of age unless stated otherwise. All mice were bred and maintained under specific pathogen-free conditions. Experiments were performed in accordance with German animal welfare legislation and approved by the relevant authorities (Regierungspräsidium Darmstadt).

### FACS analysis and sorting of HSPCs

BM cells isolated from femurs, tibias, coxae and sternum were either crushed or flushed (excluding sternum) followed by a depletion of lineage marker-positive cells (EasySep Biotin Selection Kit, Stemcell Technologies) using the following biotin-labelled antibodies (CD3, CD45R, CD19, CD11b, CD41, Ter119 and Gr1). Alternatively, BM mononuclear cells were enriched using Ficoll and then stained using the same lineage markers. Streptavidin was used to stain for the remaining lineage marker-positive cells. The cells were stained with fluorochrome-labelled antibodies and sorted using a FACS Aria I and III (BD) or analysed using a LSR Fortessa (BD). The following surface marker combinations were used to identify various HSPC populations: KL (Lin^−^ CD117^+^ Sca1^−^), LSK (Lin^−^ CD117^+^ Sca1^+^), (LT)-HSCs (Lin^−^ CD117^+^ Sca1^+^ CD150^+^ CD34^low/−^ CD48^−^), MPPs (Lin^−^ CD117^+^ Sca1^+^ CD150^−^ CD34^+^), GMPs (Lin^−^ CD117^+^ Sca1^−^ CD150^−^ CD34^+^) and MEPs (Lin^−^ CD117^+^ Sca1^−^ CD150^+^). The antibodies used are listed in Supplementary Table 1. The gating was performed as previously described^16^. Viable sorted cells were counted with trypan blue exclusion. FACS data analysis was performed with DIVA 7.0 software (BD) or FlowJo software (FlowJo).

### MiR expression array by quantitative RT–PCR

Conditional deletion of STAT5A/B was induced in 6-week-old mice (STAT5^fl/fl^ x Mx1:Cre and STAT5^fl/fl^; 16-20 mice per group and experiment) via Poly(I:C) injections as previously described^39^. LT-HSCs were isolated from 12- to 16-week-old mice via FACS sorting. 5,000–10,000 viable LT-HSCs were starved for 5 h and subsequently stimulated with 100 ng ml^−1^ TPO (Peprotech) for 2 h. The RNA was isolated by using a miRNeasy mini kit (Qiagen). cDNA was synthesized from total RNA using a miR gene-specific RT-primer pool according to the MicroRNA Megaplex Assay protocol (Megaplex Pools—Applied Biosystems). The preamplification reaction was performed according to the manufacture's protocol. The TaqMan Array MicroRNA Rodent Cards were analysed using the 384-well TaqMan Low Density Array default thermal cycling conditions (ABI PRISM 7900HT). The CT (threshold cycle) values were determined using default threshold settings. Three different housekeeping genes were used for data normalization. To identify TPO-induced miRs that are dependent on STAT5, we measured the fold expression change of miRs upregulated following TPO stimulation in the presence (STAT5^fl/fl^) and absence (STAT5^fl/fl^ x Mx1:Cre) of STAT5. All statistical analyses were performed using a two-tailed unpaired *t*-test. *N*=2 sets of 16–20 mice per experiment and group. The miR expression array data can be accessed from Figshare at: http://dx.doi.org/10.6084/m9.figshare.1554878.

### Quantitative RT–PCR of c-Kit expression

To quantify the ability of miR-193b to target c-Kit mRNA expression, FACS-sorted LT-HSCs and MPPs were lentivirally transduced for ectopic miR-193b expression or control at multiplicity of infection (MOI)=100 and cultured for 4 days. Fluorescent cells were harvested and directly lysed and reverse transcribed using the Cells-to-Ct-Kit (Life Technologies) according to the manufacturer's protocol. The pre-amplification for 14 cycles was performed according to the manual of TaqMan-PreAmp-Master-Mix (Life Technologies) using TaqMan assays for c-Kit (ID: MM00445212) and Gapdh (ID: MM099999915_g1), before qPCR was performed according to the manufacturer's protocol using the respective TaqMan assays and the Gene Expression Master Mix (Life Technologies). The data were normalized to Gapdh in each sample and displayed as Δct.

### QPCR of miR-193b basal, stress and cytokine stimulation

To assess the endogenous miR-193b levels under steady-state and under stress, LT-HSCs, MPPs, KL and Lin^+^ cells were FACS-sorted 10 days after injection of 150 mg kg^−1^ 5-FU in four individual 12-week-old *miR-193b*^*+/+*^ mice. As a reference under steady-state haematopoiesis, LT-HSCs, MPPs, KL and Lin^+^ cells were isolated from BM of 12-week-old *miR-193b*^*+/+*^ mice via FACS.

To determine the kinetics of miR-193b expression after cytokine stimulation, FACS-sorted LT-HSCs were starved for 1 h and stimulated for 0, 20, 60 and 120 min with 100 ng ml^−1^ SCF, 100 ng ml^−1^ TPO, 20 ng ml^−1^ interleukin (IL) 3, 20 ng ml^−1^ IL6, 5 U ml^−1^ EPO in SFEM (Serum-free Expansion Medium, Stemcell Technologies) at 37 °C/5% CO_2_.

Cells were lysed according to the manufacturer's protocol of the Cells-to-Ct-Kit (Life Technologies) the reverse transcription was performed with the TaqMan MicroRNA RT Kit (Life Technologies) according to the manufacturer's instructions using the mmu-miR-193b-3p (ID002467) and snoRNA202 (ID001232) assays. LT-HSCs and MPPs were pre-amplified for 12 cycles using the TaqMan PreAmp Master Mix (Life Technologies) according to the manufacturer's protocol. Reverse transcribed samples from KL and Lin^+^ cells were directly used for qPCR according to the manufacturer's instructions of the Cells-to-Ct-Kit. The TaqMan Universal Master Mix II with UNG (Life Technologies) was used for the qPCR performed on a StepOne instrument (Applied Biosystems). SnoRNA202 was used for normalization.

### Competitive repopulation assay

FACS-sorted LT-HSCs from 12-month-old *miR-193b*^*−/−*^ or *miR-193b*^*+/+*^ mice (CD45.2) were transplanted intravenously (100 LT-HSCs/mouse) into sub-lethally irradiated (2.5 Gy) 6- to 8-week-old NSG mice (CD45.1) together with 2.5 × 10^5^ BM competitor-recipient cells (CD45.1). For the miR-193b overexpression transplantation, 350 FACS-sorted LT-HSCs from 3-month-old C57.BL/6 mice (CD45.2) were lentivirally transduced 24 h prior transplantation and injected into the tail vein of lethally irradiated B6.SJL-PtprcaPepcb/BoyJ (CD45.1) recipients (6- to 8-week-old) together with 2 × 10^5^ BM competitor recipient cells (CD45.1). Transduction efficiency of the transplanted LT-HSCs was determined via FACS of a remaining cell aliquot after 3 days in culture. Multilineage reconstitution was measured every 4 to 6 weeks post transplantation in PB. Briefly, red blood cells were lysed with PharmLysis Buffer (BD), and cells were then stained with antibodies against CD45.1, CD45.2, CD3, B220, Ter119 and CD11b/Ly6G and a dead/live cell exclusion (Fixable Viability Dye, eBioscience). Lentivirally transduced haematopoietic cells were detected by their enhanced blue fluorescent protein (eBFP) expression via FACS. The primary recipients were killed 16–24 weeks after transplantation, and 1 × 10^6^ BM cells per mouse were transplanted into secondary sublethally irradiated NSG recipients (2.5 Gy) or lethally irradiated B6.SJL-PtprcaPepcb/BoyJ mice. For BM reconstitution analyses (primary and secondary recipients), ficoll gradient-enriched BM cells (Histopaque 1083, Sigma) were stained with antibodies against CD45.1, CD45.2, CD3, B220, Ter119 and CD11b/Gr1 and a dead/live cell exclusion (Fixable Viability Dye, eBioscience), as well as for CD117, Sca1, CD150, CD48, CD16/32, CD34, Lineage markers, and investigated via FACS.

### Vector construction

The third-generation self-inactivating lentiviral vector pRRL.PPT.SFFV.eGFP.wPRE (Schambach 2006) was used to construct the miR-193b and the miR-132/212 expression vectors. The open reading frame (ORF) of eGFP (enhanced green fluorescent protein) was replaced with eBFP2. The genomic region of miR-193b was amplified from splenocytes of C57Bl/6 mice using the forward primer (5′-GAGCTGTACAAGTAATAGGTGGATGGGGTGGGGTGTTT-3′) and reverse primer (5′-TAAGGTACCATTTAAATACTAGTCAGGAAGCCTTTCGGGGATG-3′). The PCR product harboured the sequence from 13449305 to 13449984 of chromosome 16. After digestion with *Bsr*GI/*Acc*65I, the genomic region including the miR-193b sequence was cloned into the *Bsr*GI site of pRRL.PPT.SFFV.eBFP.wPRE 3′ of eBFP2 (Supplementary Fig. 3a). For miR-132/212, the forward primer (5′- GAGCTGTACAAGTAATAGCCGCTGGGACATCTTTGACG-3′) and the reverse primer (5′-TAAGGTACCATTTAAATACTAGTTCCTTCTCCTCCCCCTTCAGC-3′) were used to amplify the genomic region from 74986757 to 74987630 of chromosome 11 from splenocytes of C57Bl/6 mice (Supplementary Fig. 3a). The ectopic expression level of mature miR-193b and miR-132 was confirmed via qPCR using an ABI TaqMan microRNA Assay ID002467 and ID000457 (Life Technologies).

To generate a c-KIT rescue vector, the eGFP ORF of the pRRL.PPT.SFFV.eGFP.wPRE vector was replaced by the IRES-VENUS-hImportin subunit α1 (AA2-67) and a multiple cloning site (MCS) was inserted downstream of the SFFV promoter sequence (pRRL.PPT.SFFV.IRES.VENUSnm.wPRE). The murine c-KIT ORF was amplified from the plasmid pENTR1A-ckit (a gift from Christian Brandts) with the forward (5′-CTTAACTAGTACCGCGATGAGAGG-3′) and the reverse (5′-GAATACCGGTTCTGCTCA CGCATCTTC-3′) primer pair and then cloned into the MCS of pRRL.PPT.SFFV.MCS.IRES.VENUSnm.wPRE (Supplementary Fig. 3b).

### Ki67/7-AAD staining for cell cycle and quiescence

Lineage-depleted BM cells were stained for CD117, Sca1, CD150, CD48, CD16/32, CD34 and Streptavidin. The cells were assessed for Ki67 expression and DNA content (7-AAD) according to the manufacturer's instruction and measured via flow cytometry using an LSR Fortessa, BD.

### Time-lapse imaging and cell tracking

Microscopy and tracking of LT-HSCs and their progeny was performed using a self-written computer programme (TTT) as previously described^16^,^17^ until the fate of all progeny in the third cell generation was determined. The generation time of an individual cell was defined as the time span from cytokinesis of its mother cell division to its own division. Dead cells were easily depicted by their shrunken, non-refracting and immobile appearance. Cell tracking was carried out by scientists; the current analysis did not rely on data generated by an unsupervised computer algorithm for automated tracking.

### *In vivo* 5-FU treatment

5-FU (Medac) was intraperitoneally injected into *miR-193b*^*−/−*^ and *miR-193b*^*+/+*^ mice (150 mg kg^−1^) once a week. The health status of the animals was monitored daily.

### Peripheral blood cell counts

Peripheral blood cell counts were determined from tail vein blood using a ScilVet animal blood cell counter (Scil Animal Care Company).

### *In vitro* cell proliferation assay

FACS-sorted LT-HSCs (100 cells per well) were cultured for 7 days in SFEM supplemented with 100 ng ml^−1^ murine SCF and TPO, 20 ng ml^−1^ murine IL3 and IL6 (Peprotech) and 5 U ml^−1^ human EPO (PromoKine). Viable cells were assessed using the ViaLight Plus Cell Proliferation and Cytotoxicity BioAssay Kit (Lonza) at days 3, 5 and 7 according to the manufacturer's instructions. Luminescence was measured using a Mithras LB940 luminometer (Berthold Technologies). For ectopic miR-193b expression experiments, FACS-sorted LT-HSCs (100 cells per well in 96-well format) were lentivirally transduced (MOI=100) and cultured for up to 9 days in SFEM supplemented with 100 ng ml^−1^ SCF and TPO. Viable cells were counted with Trypan blue exclusion. The percentage of transduced cells (eBFP^+^) was analysed via FACS (BD CantoII). Ectopic expression of the unrelated miRs-132/212 in LT-HSCs using the same lentiviral expression strategy served as an additional control. FACS-sorted GMPs (300 cells per well in 96-well format) were lentivirally transduced (MOI=20) and cultured for up to 7 days in SFEM supplemented with 100 ng ml^−1^ SCF and 20 ng ml^−1^ murine IL3 and IL6 (Peprotech). The percentage of transduced cells (eBFP^+^) was analysed via FACS.

### Rescue experiment with ectopic c-KIT

Freshly sorted LT-HSCs (100 cells per well) were double transduced with four different combinations of either a vector coding for murine c-KIT (without 3′-untranslated region and miR-193b target site, Supplementary Fig. 3b) or a corresponding empty vector (both co-expressing VENUS), and a miR-193b expression vector or a corresponding empty vector (both co-expressing eBFP2). A MOI 50 was used for each vector. The cells were cultured for up to 9 days in SFEM (Stemcell Technologies) supplemented with 100 ng ml^−1^ murine SCF and TPO. Reporter fluorescence was measured over time via FACS (Canto II, BD). The ratio of double transduced cells was calculated by dividing the absolute number of double transduced cells at day 9 by the initial absolute number of double transduced cells at day 3.

### *In vitro* differentiation

FACS-sorted LT-HSCs (100 cells per well) from *miR-193b*^*−/−*^ and *miR-193b*^*+/+*^ mice were cultured in SFEM (Stemcell Technologies) supplemented with 100 ng ml^−1^ murine SCF and TPO, 20 ng ml^−1^ murine IL3 and IL6 (Peprotech) and 5 U ml^−1^ human EPO (PromoKine). Cells were analysed via FACS with antibodies against CD117 and CD16/32 and a dead/live cell exclusion (Fixable Viability Dye, 0.1 μl per test, eBioscience). During myelomonocytic differentiation, all CD117^+^ cells (CD117^+^ CD16/32^−^) first start to express CD16/32 (CD117^+^ CD16/32^+^) before they lose CD117 expression (CD117^−^ CD16/32^+^).

### BrdU labelling *in vivo*

We intraperitoneally injected mice with 1.5 mg BrdU (BD) 4 h before killing the animals. Lineage-depleted BM cells were stained for CD117, Sca1, CD150, CD48, CD16/32, CD34 and streptavidin. The cells were analysed for the BrdU incorporation and DNA content (7AAD) using a FITC-labelled anti-BrdU antibody (BD) via flow cytometry (LSR Fortessa, BD) according to the manufacturer's instructions (BD).

### Virus production

Vesicular Stomatitis Virus-G-pseudotyped lentiviral particles were produced using a split genome approach via calcium-phosphate-mediated transient transfection of human embryonic kidney HEK293T producer cells as recently described^16^. After 48 h, supernatant was collected, filtered (45 μm) and enriched via ultracentrifugation (50,000 g, 2 h). Viral titres were determined via transduction of NIH3T3 cells with various concentrations of virus supernatant and FACS analysis.

### RNA sequencing

A total 10,000 LSK cells (CD117^+^ Sca1^+^ Lineage^−^) from four *miR-193b*^*−/−*^ and *miR-193b*^*+/+*^ mice were isolated via FACS. The cells of one genotype were then pooled. The RNA of two independent sets of pooled LSK cells was sequenced. The quality and concentration of the libraries were determined using an Agilent 2,100 Bioanalyser and RiboGreen fluorescence on QuBit (Life Technologies). The libraries were sequenced using a HiSeq2000 system (Illumina). Sample preparation and data analysis were performed as previously described^15^. The RNA-Seq statistical analysis was performed using Partek genomics suit 6.6 software.

### DAVID Bioinformatics Resources functional cluster analysis

The list of significantly differentially regulated genes (1.5-fold upregulated or downregulated in *miR-193b*^*−/−*^ LSK, *P*<0.05) was submitted as a list with official gene symbols for DAVID analysis and aligned with murine genetic background. The count threshold and the ‘EASE' value were set to 2 and 0.1, respectively. Functional annotation was performed via KEGG pathway analysis.

### Ingenuity pathway analysis

To obtain information about the miR-193b- dependent biological processes from the RNAseq data, gene expression data were analysed using Ingenuity Pathways Analysis with default settings according to the manufacturer's instructions (IPA) v.5.0 (Ingenuity Systems Inc). This tool provides information about diseases, molecular function and biological process categories, as well as biological pathways related to the genes obtained from the RNAseq analysis (Supplementary Data 2). In addition, IPA maps each gene within a global molecular network developed from information contained in the Ingenuity Pathways Knowledge Base. Gene networks are generated algorithmically based on their connectivity in terms of expression, activation, transcription and/or inhibition. We used IPA to identify canonical pathways that are affected by miR-193b and upstream regulators predicted to be responsible for the observed mRNA expression changes.

### Phosphoflow cytometry

To assess basal signalling activity, the BM cells were flushed in ice-cold PBS and directly fixed in Fix Buffer I after centrifugation. To examine cytokine activation, FACS-sorted LT-HSCs were starved for 1 h in SFEM at 37 °C/5% CO_2_. Next, the cells were either left untreated (control) or stimulated for 20 min with 100 ng ml^−1^ SCF and TPO, 20 ng ml^−1^ IL3 and IL6 and 5 U ml^−1^ EPO. For the stimulation kinetics, LT-HSCs were stimulated for 0, 5, 20, 60 and 120 min with the aforementioned cytokine cocktail. The cells were then fixed with Fix Buffer I (BD). After washing the cells in PBS+1% FCS+0.09% NaN_3_, they were permeabilized with ice-cold Perm Buffer III (BD) for 30 min on ice according to the manufacturer's instructions. Permeabilized cells were subsequently stained with antibodies against p-STAT5-PE, p-ERK1/2-FITC, p-AKT-BV421 and p-STAT3-AF647 (BD) to analyse the phosphorylation status of the cells via FACS. Freshly isolated BM cells stimulated with 100 ng ml^−1^ SCF and TPO, 20 ng ml^−1^ IL3 and IL6 and 5 U ml^−1^ EPO for 20 min before fixation served as a positive control.

### PamGene serine/threonine and tyrosine array

The kinase activity assessment of total BM cells was accomplished using PamChip Tyrosine and Serine/Threonine Kinase Array Chips and a PamStation 12 system according to the manufacturer's instructions. Briefly, 2 × 10^6^ total BM cells isolated from four individual male mice of each genotype (*miR-193b*^*−/−*^ and *miR-193b*^*+/+*^) were lysed in M-PER Mammalian Extraction Buffer (Pierce).

For the tyrosine kinase array, 2 μg of cleared cellular lysate was mixed with 4 μl of 10 × protein tyrosine kinase reaction buffer (PK), 0.4 μl of 1 M dithiothreitol, 0.4 μl 100 × BSA, 1 μl of 4 mM ATP and 0.3 μl 1 mg ml^−1^ monoclonal anti-phosphotyrosine FITC conjugate (clone PY20), which was adjusted to 40 μl with distilled H_2_O. All chemicals were provided by PamGene International BV. Each array was blocked with 0.2% bovine serum albumin and washed with PK solution. A kinase reaction was then carried out at 30 °C. The reaction mix was pulsed back and forth through the porous material of the PamChip for 60 cycles. A picture was taken with a built-in CCD camera every fifth cycle.

For the Serine/Threonine Kinase Array, 2 μg of cleared cellular lysate was mixed with 4 μl of 10 × PK buffer, 0.4 μl of 100 × BSA and 1 μl of 4 mM ATP adjusted to 40 μl with distilled H_2_O. Each array was blocked with 0.2% bovine serum albumin and washed with PK solution. A kinase reaction was subsequently carried out at 30 °C. The reaction mix was pulsed back and forth through the porous material of the PamChip for 60 cycles. Next, the Detection Mix consisting of 3 μl of 10 × Antibody buffer, 0.34 μl of STK antibody mix and 0.4 μl of STK antibody FITC-labelled, adjusted to 30 μl with distilled H_2_O was added to the chip. The reaction mix was pulsed back and forth through the porous material of the PamChip for an additional 30 cycles. A picture was taken with a built-in CCD camera every fifth cycle.

Spot intensities were normalized to the local background signal by subtracting the median background signal from the median spot intensity using BioNavigator (PamGene) software. A constant background of 40 was subtracted from each raw median signal. The value of 40 was assessed in a preliminary experiment from an empty array, which was not prepared with any sample. To make arrays comparable and to remove a typical mean-variance-dependency, we normalized the data using the ‘vsn' method. This method also allows for negative values and therefore uses the arcsinh instead of the logarithm for variance stabilization. All analyses were performed with R software (version 3.0.1). Comparing *miR-193b*^*−/−*^ and *miR-193b*^*+/+*^ samples revealed differentially expressed peptides using the linear models proposed by Smyth^40^, which are implemented in the R-package ‘limma'. To reduce the number of false positive results, raw *P*-values were adjusted using the method of Benjamini and Hochberg. The log2 fold change (log2 FC) was assessed to determine whether genes were upregulated or downregulated.

### Endogenous c-KIT expression determined by FACS

Total c-KIT expression was determined after fixation and permeabilization of BM cells in BD Cytofix Fixation Buffer and Perm Buffer III. Next, the cells were stained with anti-c-KIT-PE-Cy7 or the respective isotype control and analysed by FACS.

### Statistics

Statistical analysis was performed with GraphPadPrism software (version 6.0, STATCON). Statistical significance was determined via *t*-test (two-tailed, unpaired and equal variances) unless otherwise mentioned (see Figure legends). The significance level for all tests was set to *α*=5%. *P value<0.05; **P value<0.01 and ***P value<0.001.

## Additional information

**Accession codes:** The RNA-seq data have been deposited in the BioProject database under accession code PRJNA267041.

**How to cite this article:** Haetscher, N. *et al*. STAT5-regulated microRNA-193b controls haematopoietic stem and progenitor cell expansion by modulating cytokine receptor signalling. *Nat. Commun.* 6:8928 doi: 10.1038/ncomms9928 (2015).

## Supplementary Material

Supplementary Information Supplementary Figures 1-7 and Supplementary Table 1

Supplementary Data 1 STAT5-dependent microRNA expression in LT-HSCs upon Thrombopoietin stimulation. LT-HSCs were isolated from conditional STAT5 null (*Mx1::Cre x STAT5^fl/fl^*) or STAT5 WT (*STAT5 ^fl/fl^*) and stimulated with the cytokine thrombopoietin for 2h. The miRNA expression profile was assessed via quantitative miR PCR (TaqMan Array MicroRNA Rodent Cards). The fold change (log2) of stimulated versus unstimulated cells and the P values are displayed.

Supplementary Data 2 Gene expression in LSK cells of miR-193b^−/−^ and miR-193b^+/+^ mice. Expression analysis was determined via RNA sequencing, and the data were processed using Partek software. All genes that were significantly differentially expressed (1.5 fold up-regulated or down-regulated, P<0.05) are displayed.

Supplementary Data 3 KEGG pathway analysis of differentially expressed genes in LSK cells. All significantly regulated genes (41 up-regulated and 117 down-regulated) in the absence of miR-193b were subjected to DAVID analysis.

Supplementary Data 4 Ingenuity pathway analysis of differentially expressed genes in LSK cells. All significantly regulated genes (41 up-regulated and 117 down-regulated) in the absence of miR-193b were subjected to Ingenuity Pathway Analysis.

Supplementary Data 5 Basal tyrosine and serine/threonine phosphorylation in miR-193b^−/−^ and miR-193b^+/+^ bone marrow cells. Phosphorylation levels were measured using PamChip Tyrosine and Serine/Threonine Kinase Array Chips from PamGene. The data are represented as raw and normalised values.
