# Supplementary material for: STAT5-regulated microRNA-193b controls haematopoietic stem and progenitor cell expansion by modulating cytokine receptor signalling
Source: Nat Commun. 2015 Nov 25;6:8928. doi: 10.1038/ncomms9928 (PMC4674773; doi:10.1038/ncomms9928)
Supplement: Supplementary Information — Supplementary Figures 1-7 and Supplementary Table 1 [file ncomms9928-s1.pdf]

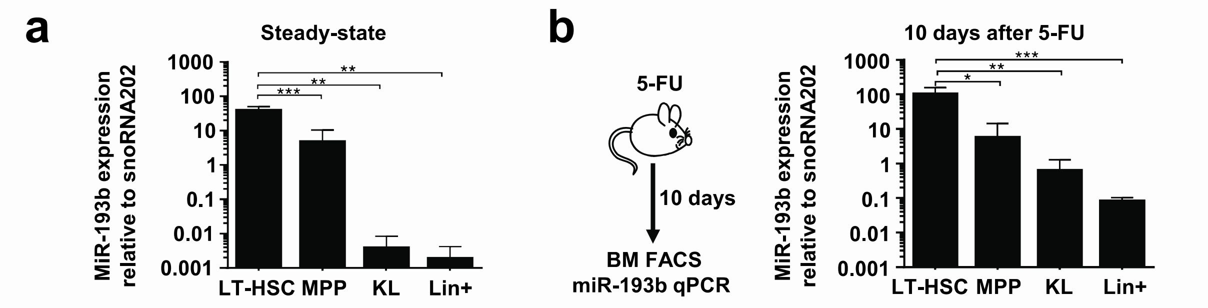


**Supplementary Figure 1. MiR-193b expression in HSPC populations in steady-state and during haematopoietic stress.** (a) Quantification of endogenous miR-193b in FACS-sorted LT-HSCs, MPPs, c-KIT$^+$ Sca1$^-$ Lin$^-$ (KL) cells and Lin$^+$ cells of adult wildtype mice in steady-state hematopoiesis, determined via miR-specific quantitative RT-PCR. MiR-193b levels were normalized to snoRNA-202 expression. (b) Quantification of endogenous miR-193b in FACS-sorted LT-HSCs, MPPs, c-KIT$^+$ Sca1$^-$ Lin- (KL) cells and Lin$^+$ cells of adult wildtype mice 10 days after a single 5-fluorouracil (5-FU) injection, determined via miR-specific quantitative RT-PCR. MiR-193b levels were normalized to snoRNA-202 expression. N=4 mice for MPPs, KL and Lin$^+$, N=3 mice for LT-HSCs. The bar diagrams represent the mean and SD. The significance was only determined between the LT-HSCs and the respective cell populations. *, $P$<0.05; **, $P$<0.01; and ***, $P$<0.001.

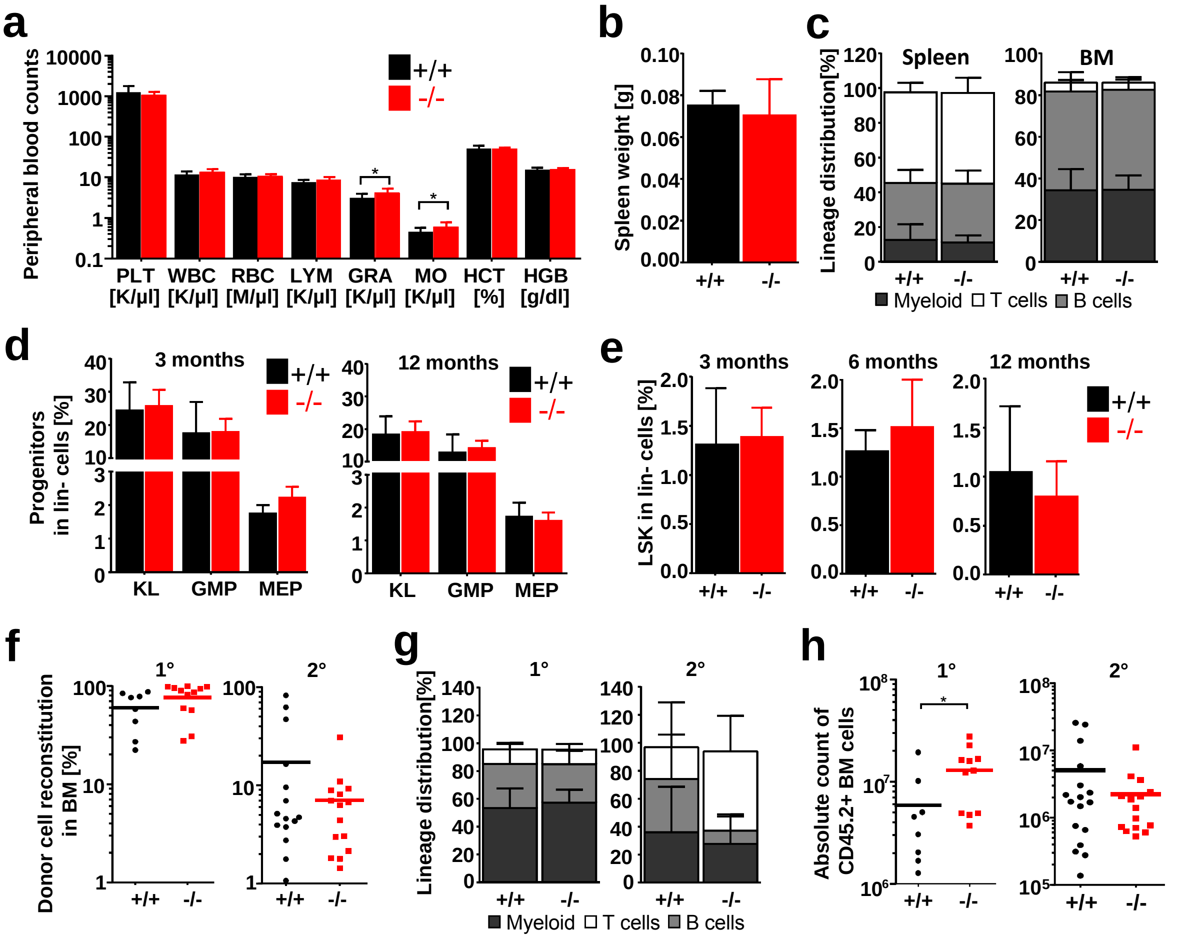


**Supplementary Figure 2. Haematopoietic cell distribution in miR-193b-deficient mice and in recipient mice transplanted with *miR-193b*$^{-/-}$ LT-HSCs.** (a) Peripheral blood counts in 3-4-month-old mice. N=11 *miR-193b*$^{+/+}$ mice, N=17 *miR-193b*$^{-/-}$ mice. (b) Spleen weights of 3-month-old mice, N=6 *miR-193b*$^{+/+}$ mice, N=14 *miR-193b*$^{-/-}$ mice. (c) Distribution of mature cell lineages in the spleen and bone marrow of 3-month-old mice, N=6 *miR-193b*$^{+/+}$ mice, N=7 *miR-193b*$^{-/-}$ mice. (d) Progenitor cell distribution in lineage enriched bone marrow in 3 months old mice, N=4 *miR-193b*$^{+/+}$ mice, N=6 *miR-193b*$^{-/-}$ mice. (e) Percentage of LSK cells in in 3-month-old mice, N=4 *miR-193b*$^{+/+}$ mice, N=6 *miR-193b*$^{-/-}$ mice. (f) Donor cell reconstitution in the bone marrow of primary and secondary transplanted recipients g) Multilineage engraftment in the bone marrow of primary and secondary transplanted recipients. (h) Absolute number of donor cells in in the bone marrow of primary and secondary transplanted recipients. Mann-Whitney test. The bar diagrams represent the mean and SD. *, $P$<0.05; **, $P$<0.01; and ***, $P$<0.001.

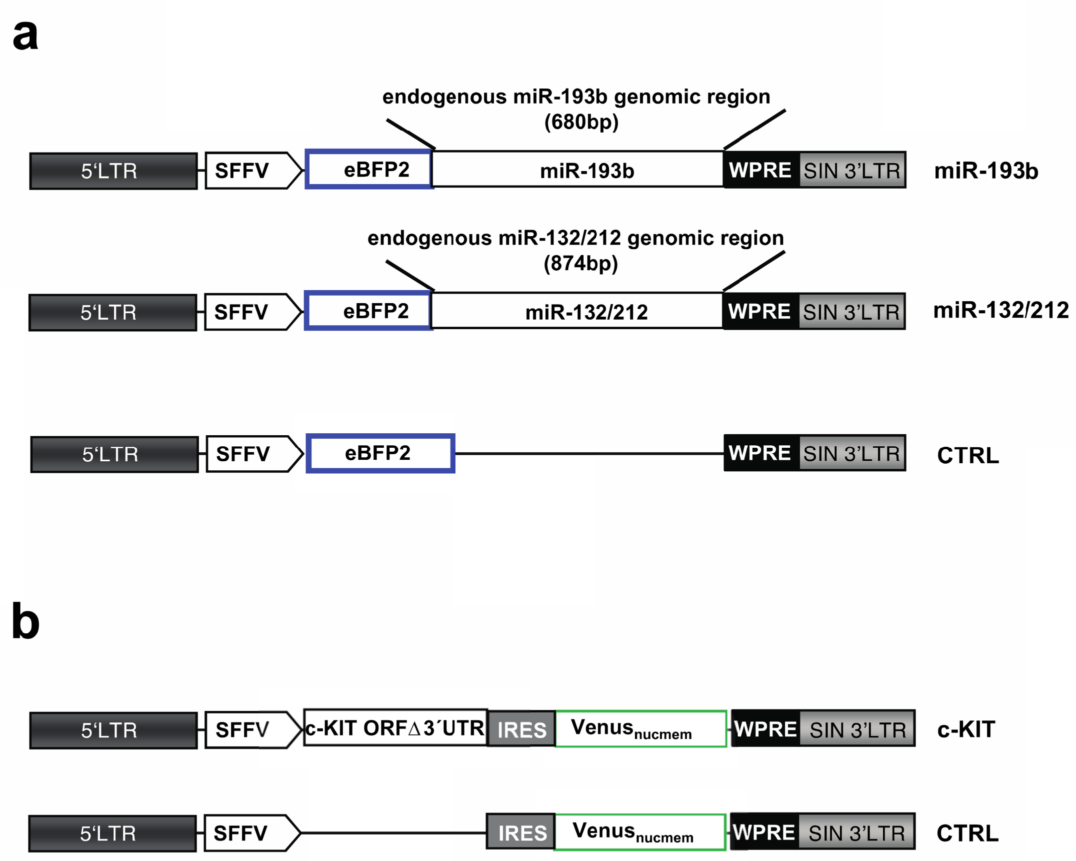


**Supplementary Figure 3. Lentiviral expression vectors.** (a) Schematic lentiviral vector maps used for ectopic miR-193b and miR-132/212 expression. The natural genomic loci encompassing the miR-193b sequence (680bp) and the miR-132/212 sequence (874bp) were cloned into the vector 3´of the open reading frame of eBFP2. (b) Schematic lentiviral vector maps used for ectopic c-KIT expression. The expression vector contains the open reading frame coding for c-KIT but not the 3´UTR containing the miR-193b targeting sequence.

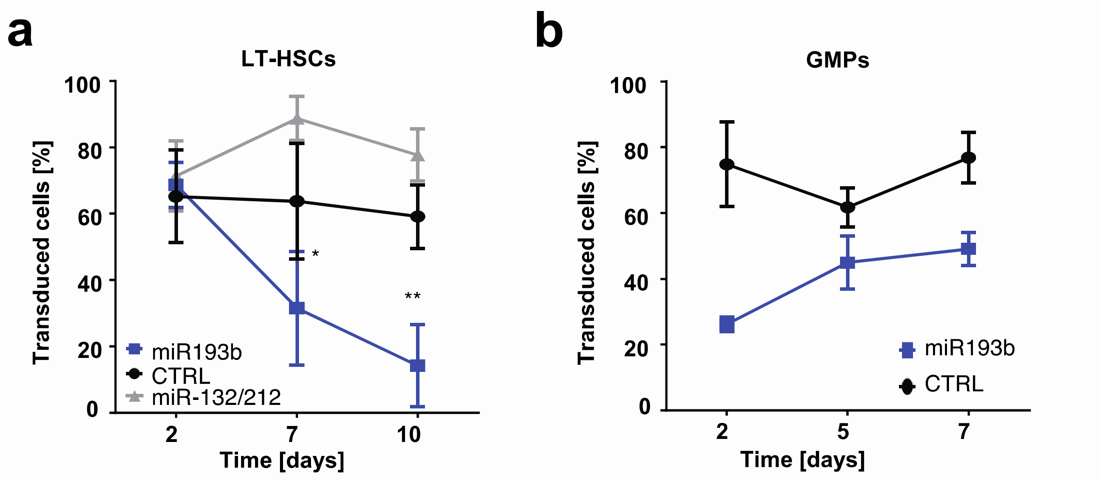


**Supplementary Figure 4. The ectopic expression of an unrelated miR-132/212 did not lead to diminished expansion of LT-HSCs.** (a) Lentiviral transduction of LT-HSCs with miR-193b, miR-132/212 or CTRL vectors and cell culture in SCF, TPO for 10 days. The percentage of transduced cells was determined via FACS at indicated time points. N=3 independent experiments. (b) Lentiviral transduction of granulocyte-macrophage progenitors (GMPs) with miR-193b or CTRL vectors and cell culture in SCF, IL3, IL6 for 7 days. The percentage of transduced cells was determined via FACS at indicated time points. N=3 independent experiments. The data is represented as mean and SD. *, $P<0.05$; **, $P<0.01$; and ***, $P<0.001$.

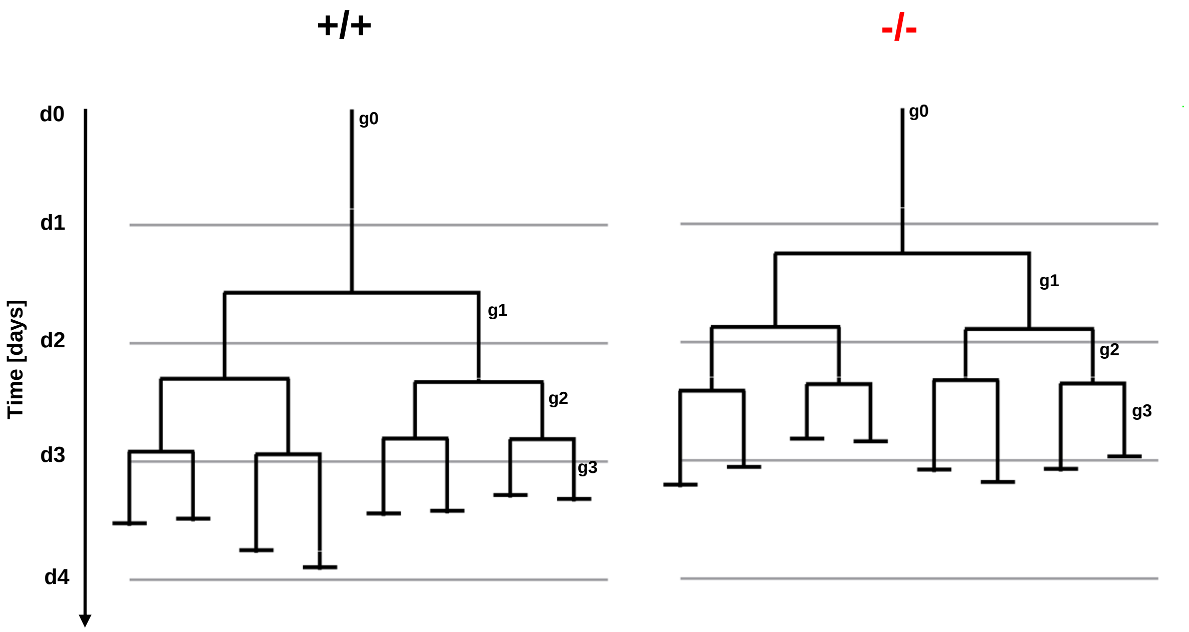


**Supplementary Figure 5. Behaviour of HSCs and their progeny determined via cell tracking.** Example pedigrees of individual HSCs and their progeny derived from *miR-193b*$^{+/+}$ and *miR-193b*$^{-/-}$ mice as assessed using video-microscopy-based cell tracking. The HSCs are represented at the apex of the pedigree; each cell division is indicated with a horizontal bar. All cells emerging from the mother HSC (g0) were tracked until generation 3 (g3).

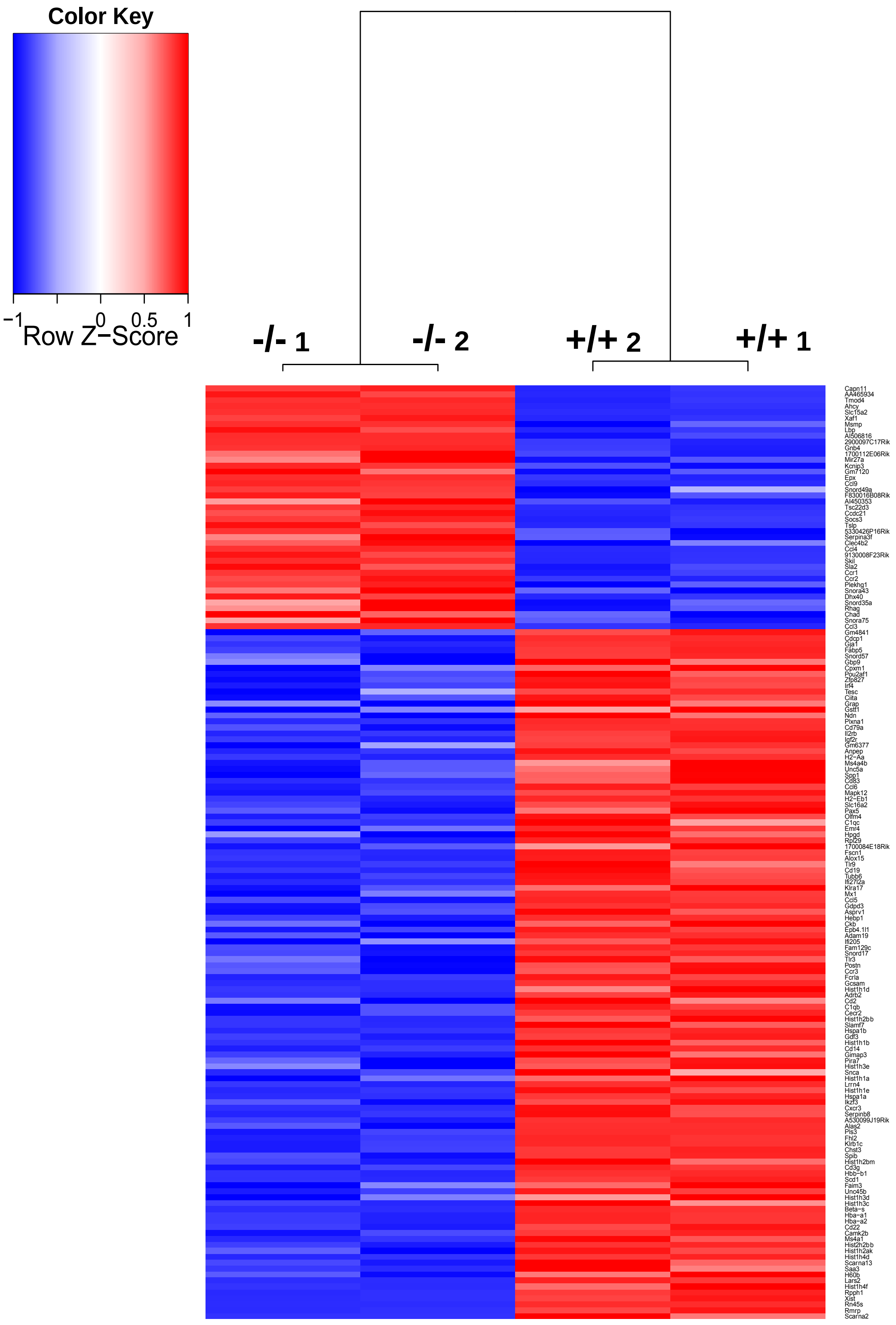


**Supplementary Figure 6. Heat map of up-regulated and down-regulated genes in *miR-193b*$^{-/-}$ LSKs.** Gene expression in LSKs from two sets of *miR-193b*$^{-/-}$ and *miR-193b*$^{+/+}$ mice was quantified using RNA sequencing. Differentially expressed genes (1.5 fold up-regulated or down-regulated, p<0.05) were clustered and displayed as a heat map.

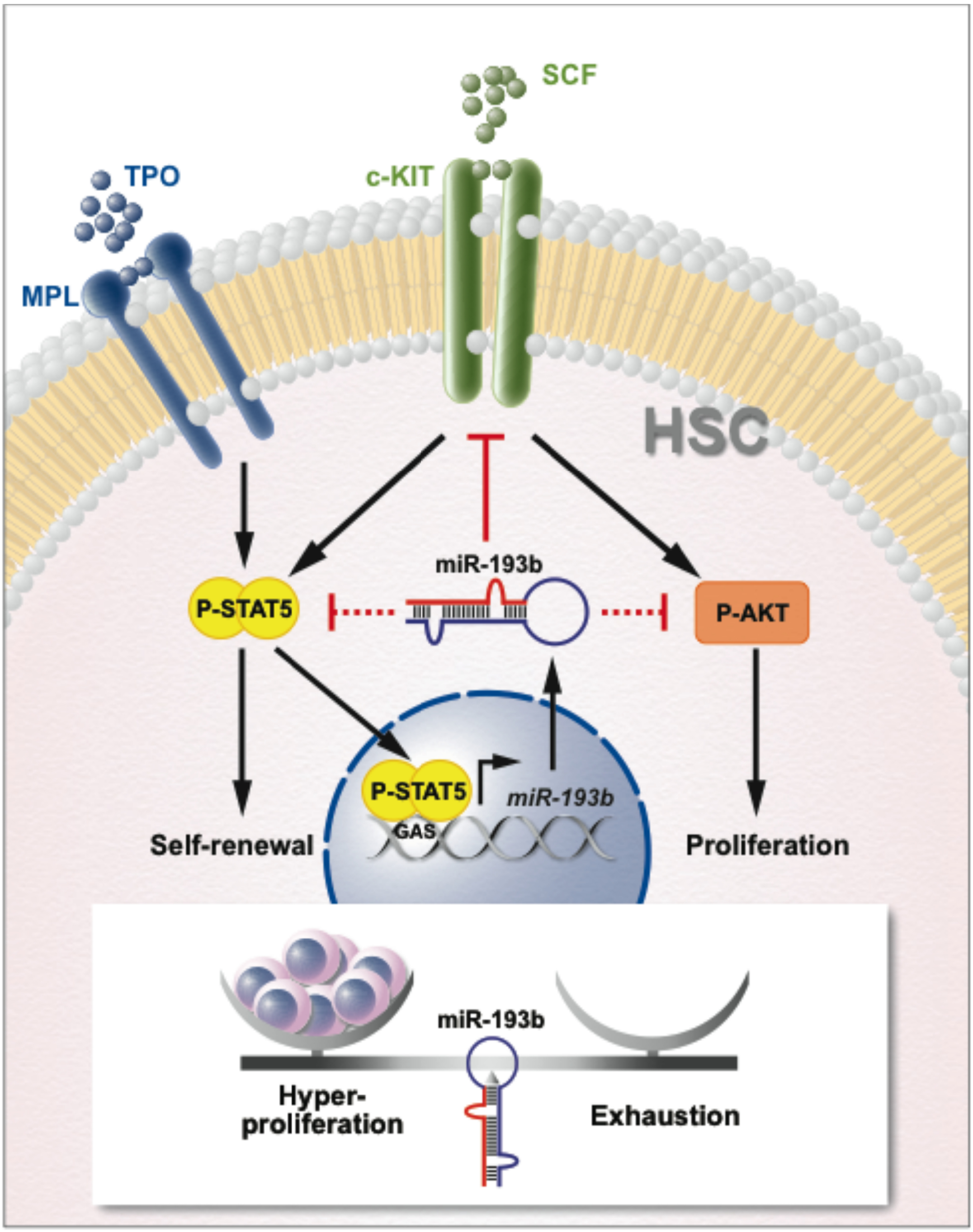


**Supplementary Figure 7. Graphical abstract of the study.** Here we show a regulatory circuit that prevents excessive HSC self-renewal by up-regulation of miR-193b driven by self-renewal promoting Thrombopoietin (TPO)–Mpl-STAT5 signalling. In turn, miR-193b restricts cytokine signalling by targeting the tyrosine kinase receptor c-KIT. The dashed lines indicate potential additional direct inhibition.

**Supplementary Table 1 Antibodies used for flow cytometry**

| Antigen | Clone | Dosage | Conjugate | Company |
|---|---|---|---|---|
| CD3 | 145-2C11 | $1\mu l/1x10^7$cells<br>1µl/test | Biotin,<br>PE-Cy7 | eBioscience |
| CD11b | M1/70 | $0.5\mu l/1x10^7$cells | Biotin | eBioscience |
| CD11b | M1/70 | 1µl/test | PE, AF670 | Biolegend |
| CD16/32 | 2.4G2 | 3µl/test | FITC, V450 | BD |
| CD19 | 1D3 | $0.5\mu l/1x10^7$cells | Biotin | eBioscience |
| CD34 | RAM34 | 25µl/test | eFluor660 | eBioscience |
| CD41 | MWReg30 | $0.5\mu l/1x10^7$cells | Biotin | eBioscience |
| CD48 | HM48.1 | 2µl/test | PE, FITC | Biolegend |
| CD45.1 | A20 | 1µl/test | eFluor450, FITC | eBioscience |
| CD45.2 | 104 | 1µl/test | PerCP-Cy5.5 | eBioscience |
| CD45R<br>(B220) | RA3-6B2 | $0.5\mu l/1x10^7$cells<br>1µl/test | Biotin,<br>PE | eBioscience |
| CD117<br>(c-KIT) | 2B8 | 3µl/test | PE-Cy7, APC,<br>BV421 | eBioscience |
| CD150 | TC15-12F12.2 | 3µl/test | PE | Biolegend |
| Sca-1 | D7 | 2µl/test | PerCP-Cy5.5,<br>BV510 | eBioscience |
| Gr-1 | RB6-8C5 | $0.5\mu l/1x10^7$cells | Biotin, | eBioscience |
| Ly6G | 1A8 | 1µl/test | PE, AF647 | Biolegend |
| Ter119 | TER-119 | $2\mu l/1x10^7$cells<br>1µl/test | Biotin<br>APC-eFlour780 | eBioscience |
| Streptavidin | | $2\mu l/1x10^7$cells | APC-eFluor780 | eBioscience |
| P- STAT5 | 47 | 20µl/test | PE | BD |
| P-STAT3 | 4 | 20µl/test | AF647 | BD |
| P-AKT | M89-61 | 5µl/test | BV421 | BD |
| P-ERK1/2 | 20A | 20µl/test | Alexa Fluor488 | BD |
| Ki67 | SolA15 | 1 µl/test | FITC | eBioscience |
| 7-AAD | | 20µl/test | | BD |
| BrdU | BU20A | 1µl/test | PE | BD |
